# Peripheral blood mononuclear cells inhibit proliferation and promote apoptosis of HeLa cells following stimulation with Bacillus Calmette-Guerin

**DOI:** 10.3892/etm.2012.855

**Published:** 2012-12-10

**Authors:** XIAOQING LU, LINGJIAO WU, ZHUO LIU, LIPING XIE, SHUO WANG

**Affiliations:** 1 Department of Surgical Urology, The First Affiliated Hospital, Zhejiang University School of Medicine, Hangzhou 310003; 2 State Key Laboratory for Diagnosis and Treatment of Infectious Diseases, The First Affiliated Hospital, Zhejiang University School of Medicine, Hangzhou 310003;; 3 Department of Surgical Urology, Zhejiang Cancer Hospital, Hangzhou 310022, P.R. China

**Keywords:** Bacillus Calmette-Guerin immunotherapy, Bacillus Calmette-Guerin-activated killer cells, peripheral blood mononuclear cells, HeLa cells, human papilloma virus-E7, retinoblastoma/E2F1 pathway

## Abstract

Bacillus Calmette-Guerin (BCG) immunotherapy is established as an effective adjuvant intravesical treatment for non-muscle invasive bladder cancer. BCG is also effective in the treatment of Condylomata acuminata caused by low-risk human papilloma virus (HPV). The aim of this study was to determine the efficacy of BCG for the treatment of cervical cancer or HPV high-risk infections. BCG-activated killer (BAK) cells were incubated with a high-risk HPV18-infected cervical cancer cell line, HeLa. The cell cycle distribution and apoptotic index of the HeLa cells were analyzed by flow cytometry. The alterations of HPV-E7, retinoblastoma (RB) and E2F1 levels were detected at the transcriptional and translational levels. The BAK cell cytotoxicity to HeLa cells was 24.08, 14.74 and 6.8% and the natural killer (NK) cell cytotoxicity was 17.62, 10.78 and 5.8% at the E/T ratios of 40:1, 20:1 and 10:1, respectively. The BAK cells significantly induced the apoptosis of HeLa cells to result in an apoptosis level of 24.2% compared with 13.45% by the NK cell treatment at the ratio of 20:1. BAK cells inhibit the proliferation of HeLa cells by G_1_/S cell cycle arrest and this may be associated with the RB/E2F1 pathway. However, G_1_/S arrest and the alteration of RB protein (pRB) and E2F1 levels in the HeLa cells did not show significant differences between the BAK cell- and NK cell-treated groups. HPV-E7 appeared not to be associated with the alteration in cell cycle progression. This study showed that immunotherapy may be a potential treatment for cervical cancer and that BCG immunotherapy may be an alternative and effective method, but further experiments and clinical trials are required to verify this effect.

## Introduction

Adjuvant intravesical Bacillus Calmette-Guerin (BCG) therapy is a well-established and successful immunotherapy for preventing local recurrences and tumor progression following the transurethral resection of non-muscle invasive bladder cancer (1,2). While the mechanism of BCG therapy remains unclear, natural killer (NK) cells play an important role in BCG-mediated antitumor effects (3). *In vitro* experiments have demonstrated that BCG-activated killer (BAK) cells, which are generated from peripheral blood mononuclear cells (PBMCs) stimulated with BCG, are the main effector cells. The BAK cell activity has been attributed to a small subpopulation of activated lymphocytes, which belong to the CD3^−^/CD8^+^/CD56^+^ NK cell phenotype (4). The BAK cells kill cancer cells mainly via perforin-mediated mechanisms rather than by Fas-FasL interactions (5).

Previous clinical studies have demonstrated that topical BCG is highly effective in the treatment of Condylomata acuminata (6,7), including flat condyloma of the cervix (8). While Condylomata acuminata is associated with low-risk human papillomavirus (HPV) infection, no study has examined the efficacy of BCG immunotherapy in high-risk HPV-related diseases such as cervical cancer. The HPV early proteins E6 and E7 are the major viral oncoproteins that regulate cell proliferation in high-risk HPV-infected cancer cells through the inactivation of the p53 and retinoblastoma (RB) tumor suppressor proteins, respectively. The RB/E2F1 pathway is a vital regulator of cell proliferation, differentiation, senescence and apoptosis (9). It has been reported that altered RB protein (pRB) expression is an independent predictor of recurrence and progression in patients treated by intravesical BCG (10), and pRB underexpression is predictive of nonresponse and cancer recurrence (11). The aim of the present study was to determine whether BCG immunotherapy has an antitumor effect on high-risk HPV infected cells, such as the HeLa cell line, and whether BCG immunotherapy alters the RB/E2F1 pathway in the HeLa cells.

## Materials and methods

### Cervical cancer cells

The established HeLa cell line (ATCC CCL-2) was used as the cervical cancer cells in the present study. The HeLa cells were grown in RPMI-1640 medium supplemented with 10% fetal bovine serum (FBS), 100 U/ml penicillin and 100 *μ*g/ml streptomycin (Gibco, Grand Island, NY, USA). The cells were incubated at 37°C in a humidified atmosphere containing 5% CO_2_.

### Isolation and stimulation of PBMCs

PBMCs from the EDTA-mediated anticoagulated blood of six informed healthy human donors were obtained using Lympholyte-H (Cedarlane, Burlington, ON, Canada) density centrifuging. The isolated PBMCs were washed twice with PBS and adjusted to a concentration of 1×10^6^ cells/ml in RPMI-1640 medium containing 10% FBS. The 50 *μ*g/ml reconstituted lyophilizate of BCG (Connaught substrain, ImmuCyst; Sanofi Pasteur, Toronto, Canada) was added to the PBMCs and the cells were cultured in six-well plates at 37°C and 5% CO_2_ for 5 days to generate BAK cells (12). Unstimulated cultured PBMCs, which are equivalent to NK cells, served as the negative controls. After 5 days, the suspended BCG-stimulated PBMCs were collected and adjusted to a concentration of 2×10^6^ cells/ml as effector cells, while the unstimulated PBMCs served as NK cells and were prepared similarly to act as the control. The study was approved by the ethics committee of the First Affiliated Hospital of Zhejiang University.

### Cytotoxicity assay

The cytotoxicity of BAK and NK cells against HeLa cells was assessed by the CellTiter 96^®^ AQueous One Solution Cell Proliferation assay (Promega, Madison, WI, USA). The HeLa cells were suspended at a concentration of 1x10^5^ cells/ml. The effector (E) and target (T) cells were combined at E/T ratios of 40:1, 20:1 and 10:1 in 96-well plates with a total volume of 200 *μ*l in each well. Combinations of E and T cells are referred to as ET. The E and T cells were cultured in RPMI-1640 medium alone to determine the spontaneous release, and the wells with 200*μ*l RPMI-1640 medium were blank (B) wells. Each group had three parallel replicate wells. After 20 h of incubation in a humidified 37°C incubator with 5% CO_2_, 20 *μ*l MTS solution was added to each well and incubated for 3.5 h. The optical density value of each well was measured at 490 nm with an automatic ELISA reader. The average value of the three wells in each group was used. The cytotoxicity was calculated as follows: Cytotoxicity (%)=[1-(OD_ET_ - OD_E_)/(OD_T_ - OD_B_)]x100%.

### Cell apoptosis assay

HeLa cells (1×10^5^ cells/ml) were incubated in 12-well plates with a volume of 1 ml in each well. After 6 h, when the cells were adherent, BAK cells (2×10^6^ cells/ml, 1 ml) were added to the adherent HeLa cells (2 ml total per well) and incubated for 20 h. Unstimulated PBMCs were added to HeLa cells at the same E/T ratio to serve as the negative control. The wells containing HeLa cells without effector cells were supplemented with 1 ml RPMI-1640 medium to serve as the blank control. Following incubation, the suspended cells (BAK and NK cells) were washed three times with PBS to remove all effector cells. The HeLa cells were trypsinized and washed with PBS, then collected and stained using the FITC Annexin V Apoptosis Detection kit I (BD Biosciences, San Diego, CA, USA) according to the manufacturer’s instructions. The stained HeLa cells were analyzed by fluorescent-activated cell sorting (FACS) using a BD LSR II Flow Cytometer (BD Biosciences).

### Cell cycle assay

The HeLa cells were incubated with the BAK or NK cells at an E/T ratio of 20:1 in 12-well plates for 20 h. Untreated HeLa cells served as the blank control. After removing the suspended effector cells with PBS, the HeLa cells were trypsinized and washed with PBS and suspended in PBS. Ethanol was added to a final concentration of 70% and the suspension was stored at 4°C overnight. The cells were washed with PBS to remove ethanol and were then suspended in PBS containing 0.25 mg/ml DNase-free RNase (Sigma-Aldrich, St. Louis, MO, USA). After nuclear staining with propidium iodide (PI, 50 *μ*g/ml; Sigma-Aldrich) in the dark at room temperature for 30 min, flow cytometry was performed using the BD LSR II Flow Cytometer system with FACSDiva software (BD Biosciences). The data from three identical analyses were used to confirm the results.

### Real-time RT-PCR

HeLa cells were incubated with BAK or NK cells at an E/T ratio of 20:1 in 12-well plates for 20 h. Untreated HeLa cells served as a blank control. After removing the suspended effector cells with PBS, total RNA was extracted from the HeLa cells (treated or untreated) using the TRIzol (Invitrogen, Carlsbad, CA, USA) method. The mRNAs were resuspended in RNase-free water. The index of purity of the mRNA samples ranged between 1.8 and 2.0 by 260/280 measurement. Total RNA was used to generate cDNA with the PrimeScript II 1st Strand cDNA Synthesis kit (Takara, Otsu, Japan) according to the manufacturer’s instructions. This was followed by detection of PCR products with iQ^™^ SYBR® Green Supermix (Bio-Rad, Hercules, CA, USA) real-time RT-PCR with primers specific for the HPV-E7, RB and E2F1 transcripts with an internal amplification control of GAPDH. The nucleotide sequences of the primers are shown in Table I. The HPV-E7, RB and E2F1 mRNA expression levels were measured using the Ct (cycle threshold) method, and relative fold-expression changes were normalized to GAPDH mRNA using the equation 2^−ΔΔCt^.

### Western blotting

The HeLa cells were incubated with BAK or NK cells at an E/T ratio of 20:1 in 12-well plates for 20 h. Untreated HeLa cells served as a blank control. Effector cells were removed with cold PBS and the remaining HeLa cells were lysed by placing on ice in cell lysis buffer (Cell Signaling Technology, Inc., Beverly, MA, USA). Cell lysates were incubated on ice for 30 min and centrifuged at 12,000 x g for 10 min at 4°C. The proteins were applied to 8–12% gels and separated by SDS-PAGE. The samples were then transferred to a polyvinylidene difluoride (PVDF) transfer membrane (Bio-Rad) for 1 h. The membranes were blocked for 2 h at room temperature with 5% dry milk in Tris-buffered saline with Tween (TBST) and incubated overnight at 4°C with primary antibodies against RB (1:1,500), E2F1 (1:3,000; Epitomics, Burlingame, CA, USA) and HPV18-E7 (1:1,000; Abcam, Cambridge, UK). Protein levels were normalized to total GAPDH using a mouse anti-GAPDH monoclonal anti-body (Abcam). The membranes were washed in TBST and incubated with 1:5,000 anti-mouse or anti-rabbit IgG conjugated to horseradish peroxidase for 1 h at room temperature before washing again. Band signals were visualized using chemiluminescence reagents (Millipore, Billerica, MA, USA), acquired in the linear range of the scanner and analyzed using QUANTITY ONE software (Bio-Rad).

### Statistical analysis

All data are presented as the median ± range. The statistical significance between treatment and control groups was determined using the Wilcoxon signed-rank test and SPSS 18.0 software (SPSS Inc., Chicago, IL, USA). P<0.05 was considered to indicate a statistically significant result.

## Results

### Cytotoxicity of BAK and NK cells

**Figure 1.**
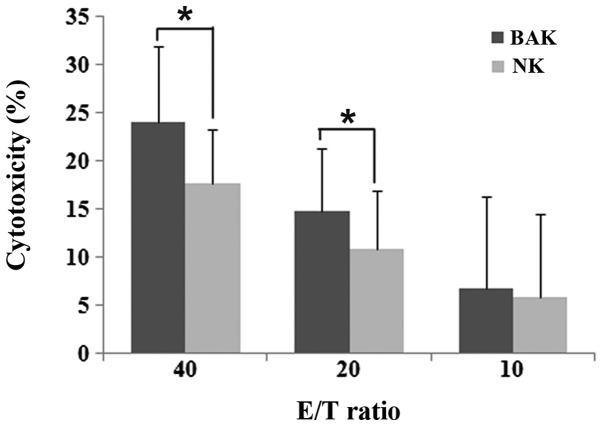
Cytotoxicity of BAK and NK cells against HeLa cells. The BAK cell cytotoxicity was 24.08±7.81, 14.74±6.61 and 6.8±9.44% and the NK cell cytotoxicity was 17.62±5.59, 10.78±6.18 and 5.8±8.7% at the E/T ratios of 40:1, 20:1 and 10:1, respectively. Between the BAK and NK groups, there was no significant difference at the ratio of 10:1 (P= 0.249). However, the cytotoxicity of the BAK cells was significantly increased at the ratios of 40:1 (P=0.028) and 20:1 (P=0.046). The cytotoxicity of the BAK and NK cells increased as the E/T ratios increased. Data are shown as the median ± range,^*^P<0.05. BAK, BCG-activated killer; NK, natural killer; E, effector; T, target cell; BCG, Bacillus Calmette-Guerin.

The PBMCs from six healthy human donors cultured with or without BCG were tested for cytotoxicity against HeLa cells by the MTS assay. The PBMCs showed increased cytotoxicity following 5 days of incubation with BCG (Fig. 1). The PBMCs stimulated with BCG (BAK cells) demonstrated higher cytotoxicity than the unstimulated PBMCs (NK cells) at the E/T ratios of 40:1 and 20:1. At the ratio of 10:1, no significant difference in cytotoxicity was observed between the BAK and NK groups (P>0.05). The cytotoxicities of the BAK and NK cells increased as the E/T ratios increased.

### Effect of BAK and NK cells on the apoptosis of HeLa cells

**Figure 2.**
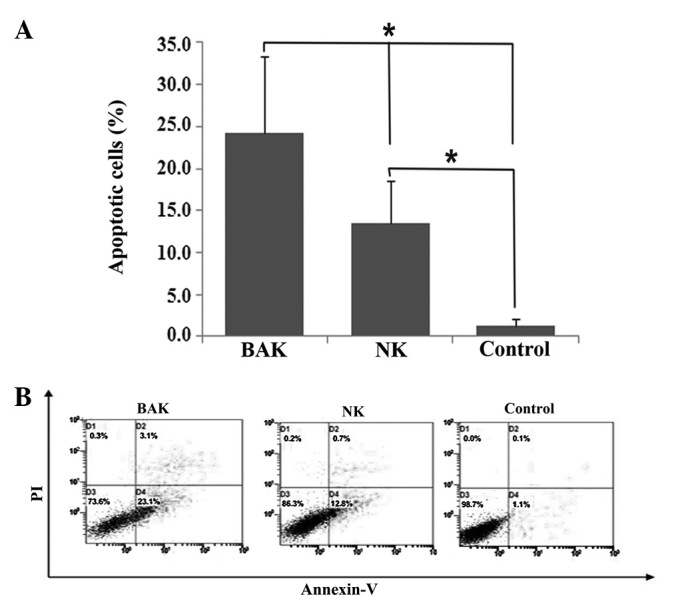
Apoptotic rates of HeLa cells following incubation with BAK and NK cells. HeLa cells without treatment were used as a blank control. Early and late apoptotic cells were combined to calculate the percentage of cell apoptosis. (A) BAK cells had a significant impact on apoptosis in HeLa cells with 24.2±9.2% apoptotic cells compared with the NK control (13.45±5.1%) and the blank control (1.25±0.8%). The NK group also showed significant apoptosis compared with the blank control (^*^P<0.05). (B) Cell apoptosis was evaluated by using FITC Annexin V/propidium iodide-double staining, and the stained HeLa cells were analyzed by fluorescent-activated cell sorting (FACS). The figure shown is representative of a set of experiments. BAK, BCG-activated killer; NK, natural killer; PI, propidium iodide; BCG, Bacillus Calmette-Guerin.

Post-incubation HeLa cells were analyzed for effector cell effects on apoptosis by flow cytometry. Although the blank controls exhibited 1.25% apoptosis and the NK cells exhibited 13.45% apoptosis, BAK cells had a significant impact on the level of apoptosis (24.2%). NK cells also showed a significant effect on the apoptosis of HeLa cells compared with the blank control (Fig. 2, P<0.05). This result suggested that PBMCs promote apoptosis of HeLa cells following stimulation with BCG.

### Effect of BAK and NK cells on the cell cycle of HeLa cells

**Figure 3.**
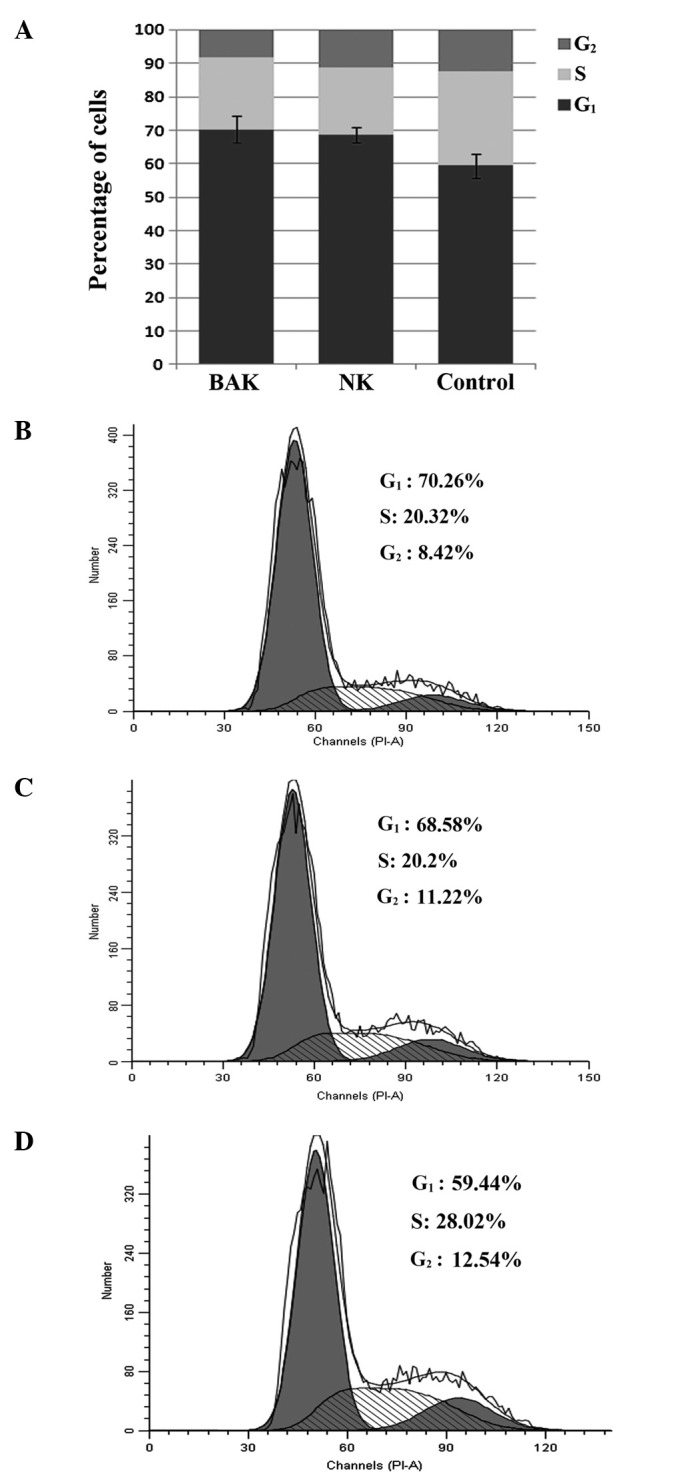
Effect of BAK and NK cells on the cell cycle in HeLa cells. (A) Compared with the normal cell cycle distribution of 59.4% in G_1_, BAK and NK cells induced G_1_/S arrest, resulting in 70.3 and 68.6% of cells, respectively, in G_1_ (P<0.05) after the incubation. However, the difference between the BAK and NK groups was not significant (P>0.05). (B–D) Cell cycle distribution of HeLa cells at 20 h after treatment with BAK or NK cells. The Y axis shows cell number. Twenty hours after co-incubation, HeLa cells were subjected to flow cytometry. (B) Incubation with BAK cells for 20 h. (C) Incubation with NK cells for 20 h. (D) Normal growth of HeLa cells for 20h.. BAK, BCG-activated killer; NK, natural killer; BCG, Bacillus Calmette-Guerin.

Post-incubation HeLa cells were analyzed for effector cell effects on the cell cycle by flow cytometry. Incubation of effector and HeLa cells induced a shift in cell cycle arrest with enhanced G_1_ phase arrest (Fig. 3). Compared with the normal HeLa cell cycle distribution where 59.4% of cells are in G_1_, BAK cells increased the level of G_1_/S arrest to 70.3% (P<0.05) and NK cells increased it to 68.6% (P<0.05). While BAK and NK cells each had a statistically significant effect on HeLa cell cycle distribution, no significant difference was observed between the BAK and NK groups (P>0.05). This result showed that PBMCs may inhibit the proliferation of HeLa cells independently of BCG stimulation; BAK cells did not exert a greater effect than NK cells.

### mRNA expression of RB, E2F1 and HPV-E7 in HeLa cells following treatment with BAK and NK cells

**Figure 4.**
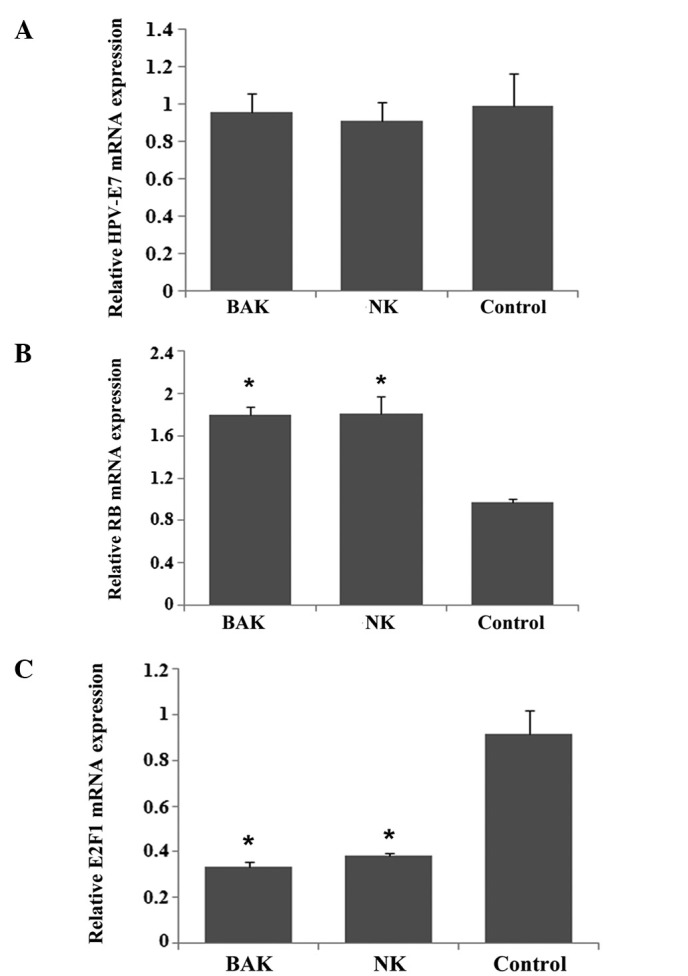
mRNA expression levels of RB, E2F1 and HPV-E7 in HeLa cells following treatment with BAK and NK cells. (A) HPV-E7 mRNA expression was very similar among BAK, NK and control groups. (B) RB mRNA expression by HeLa cells increased following treatment with BAK or NK cells, but no significant difference was observed between the two groups (P>0.05). (C) E2F1 mRNA expression decreased almost 3-fold after treatment with BAK or NK cells, but there was also no significant difference between the BAK and NK groups (P>0.05).^*^P<0.05, compared with the blank control. RB, retinoblastoma; HPV, human papillomavirus; BAK, BCG-activated killer; NK, natural killer; BCG, Bacillus Calmette-Guerin.

The HPV-E7 mRNA expression levels were very similar in the BAK, NK and control groups (Fig. 4A). RB mRNA expression in the HeLa cells increased following treatment with either BAK or NK cells (Fig. 4B, P<0.05), but no significant difference existed between the two groups (P>0.05). The E2F1 mRNA expression showed the opposite result compared with RB; E2F1 mRNA expression was reduced almost 3-fold following treatment with BAK or NK cells (Fig. 4C, P<0.05). However, there was also no significant difference between the effects of BAK and NK cells on the expression levels of either RB or E2F1. RB and E2F1 are associated with the cell cycle, therefore, this result was consistent with the results of the cell cycle assay.

### Expression of RB, E2F1 and HPV-E7 proteins in HeLa cells following treatment with BAK and NK cells

**Figure 5.**
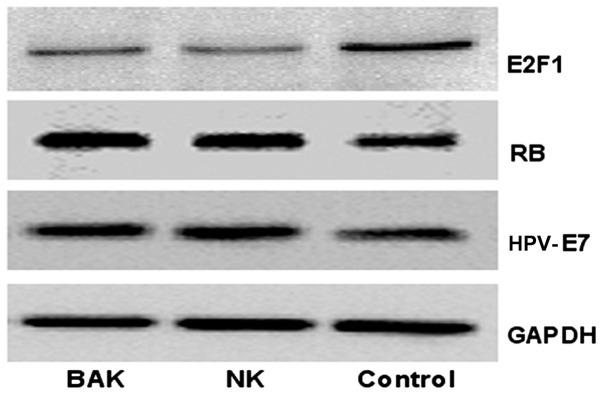
Expression of RB, E2F1 and HPV-E7 proteins in HeLa cells following treatment with BAK and NK cells. The expression of RB and HPV-E7 proteins in HeLa cells increased in BAK and NK groups compared with the blank control group. By contrast, the expression of E2F1 protein decreased after the incubation. However, the change of the three proteins between the BAK and NK groups was not significant. Data shown are a representative set of the experiments. RB, Retinoblastoma; HPV, human papillomavirus; BAK, BCG-activated killer; NK, natural killer; BCG, Bacillus Calmette-Guerin.

The RB and HPV-E7 protein expression levels increased in HeLa cells following treatment with BAK and NK cells compared with their levels in the blank control. By contrast, the E2F1 protein expression level decreased. However, the differences in RB, E2F1 and HPV-E7 protein expression levels between the BAK and NK cell groups were not significant (P>0.05) (Fig. 5). The results concerning the RB and E2F1 proteins were also consistent with the cycle assay, and the result showed that the transcription and translation of RB and E2F1 were consistent.

## Discussion

Intravesical BCG immunotherapy is a well-established treatment for human bladder cancer and is commonly used as the first-line adjuvant treatment. The mechanism of BCG immunotherapy is complex and remains unclear, however, it is mainly dependent on the activation of a number of immunocytes (including macrophages, NK cells, CD4^+^ and CD8+ T cells) and cytokines (including IFN-γ, IL-2, IL-12, TNF-α and TNF-β) (13). NK cells are essential for effective BCG immunotherapy (3). BAK cells have the CD3^−^/CD8^+^/CD56^+^/CD16^+^ phenotype of a subpopulation of NK cells and possibly NK T lymphocytes (4). It has been reported that topical BCG for the treatment of genital warts attained a high success rate (6,7), even in flat condyloma of the cervix (8). Condylomata acuminata (genital warts) are caused by HPV. Infection with oncogenic HPV is the leading cause of cervical carcinoma. In this study, we investigated the feasibility of BCG immunotherapy for the treatment of high-risk HPV-infected cervical cancer by examining its cytotoxicity on HeLa cells. The HeLa cell line is an immortal cervical cancer cell line which is infected with HPV18. PBMCs stimulated with BCG have been shown to generate BAK cells in a previous study (12).

In the current study, PBMCs stimulated with BCG showed more cytotoxicity against HeLa cells compared with PBMCs that were not stimulated with BCG. We observed that the apoptotic cell ratio was significantly higher in the BAK group than in the NK and control groups. However, it was also shown that BCG-stimulated and untreated PBMCs were able to increase the apoptotic index of HeLa cells, although the effect was more pronounced for the BCG-stimulated PBMCs. Perforin and Fas ligand (FasL) are the major cytolytic molecules of cytotoxic lymphocytes (14). The cellular mediators of BCG effector mechanisms kill targets via perforin and independently of the FasL pathway. BCG-activated lymphocytes express higher levels of perforin (5), and this may be the mechanism of the increased apoptosis observed in HeLa cells.

We investigated whether any associated influence on HPV-E7 protein expression and the RB/E2F1 pathway results from the BCG treatment. The high risk HPVs (such as HPV-16 and HPV-18) that are associated with specific anogenital cancers encode two oncoproteins E6 and E7, which are expressed in HPV-positive cancers. High-risk HPV-E7 is a major oncoprotein that plays a crucial role in the development of cervical cancer. The HPV-E7 protein functions in cellular transformation via interactions with pRB (15). The important roles of RB have been demonstrated in the suppression of cellular proliferation (16), stimulation of differentiation and senescence (17,18), cellular survival (19) and the maintenance of stem cell quiescence (20). RB plays a key role in the regulation of cell cycle progression and it is essential for the proper modulation of G_1_/S transition. pRB exerts its cell cycle regulatory functions mainly by targeting the E2F family of transcription factors and has been shown to physically interact with E2F1, 2 and 3, repressing their transcriptional activity. Multiple genes involved in DNA synthesis and cell cycle progression are regulated by E2Fs, and RB prevents their expression by inhibiting E2F activity and thereby inducing growth arrest (9).

In the present study, flow cytometric cell cycle analysis showed that HeLa cells treated with BAK or NK cells demonstrated a shift in the cell population from G_1_ to S arrest. However, BAK cells did not show a more pronounced effect than NK cells on the G_1_/S arrest. It is known that RB plays a major role in the regulation of cell cycle progression, it is essential for the proper modulation of G_1_/S transition. We measured the changes of RB and E2F1 at the transcriptional and translational levels. The results of quantitative real-time PCR (qRT-PCR) and western blotting showed consistent changes in RB and E2F1. BAK cells may suppress E2F1 expression in HeLa cells by increasing the expression of RB. E2F1 is a member of the E2F family of transcription factors and plays a crucial role in the cell cycle during the G_1_/S transition. This may be the reason why HeLa cells arrest at G_1_/S following incubation with BAK and NK cells. However, the effect on RB and E2F1 was not significantly different between the BAK and the NK groups.

It has been established that inactivation of RB by interaction with HPV-E7 leads to a release of the repression of E2F activity by RB, and thereby facilitates cell cycle progression (21). We also hypothesized that RB/E2F1 pathway alteration was associated with HPV-E7. We predicted that HPV-E7 protein expression would decrease partly to activate more RB in order to suppress E2F1 in the BAK cell-treated HeLa cells. However, the HPV-E7 mRNA expression presented at a consistent level among the BAK, NK and blank control groups. The expression level of HPV-E7 protein in the BAK and NK groups was slightly higher than in the blank control in which the HeLa cells were not treated with effector cells. In addition, HPV-E7 protein levels were not significantly different between the BAK and NK cell-treated groups. The HPV-E7 protein was previously supposed to be decreased in the HeLa cells following treatment with BAK or NK cells. By contrast, it was increased in our study. The reason may be that HPV-E7 viral DNA was randomly integrated into the host genome and increased following the overexpression of certain proteinases during the progress of apoptosis. We suggest that HPV-E7 proteins were inactive, and they could not be combined with RB proteins or that the expression level of HPV-E7 was less than that of pRB. We conclude that the BAK or NK cells may affect the RB/E2F1 pathway during the process of killing the HeLa cells by increasing the expression of RB and reducing the expression of E2F1, but the alterations of pRB and E2F1 were not correlated with the expression of HPV-E7 protein.

In addition, it has been reported that altered pRB expression is an independent predictor of the recurrence and progression of non-muscle invasive bladder cancer following BCG treatment (10). The nuclear pRB underexpression may be predictive of nonresponse and cancer recurrence following intravesical BCG+IFN-α therapy (11). Genetic alterations of the RB gene and aberrant post-translational modifications of the RB protein have also been implicated in invasive bladder cancer. Alterations in the RB gene or protein are becoming candidate targets for novel therapeutics (22). In the current study, changes of RB transcription and translation were detected in HeLa cells following treatment with BAK or NK cells. We consider that the immunotherapy may be an effective therapy for cervical carcinoma based on this study, and BCG immunotherapy, which promoted apoptosis of target cancer cells, is an alternative method.

In summary, our study demonstrates that PBMCs inhibit the proliferation of the human cervical carcinoma cell line, HeLa, by G_1_/S arrest and the promotion of apoptosis of HeLa cells following stimulation with BCG. The mechanism of G_1_/S arrest may be correlated with the RB/E2F1 pathway, but the RB and E2F1 alterations are not caused by HPV-E7. This study showed that BCG immunotherapy is a potential treatment for cervical cancer. Our study is limited and preliminary, and further experiments and clinical trials are required to verify this effect.

## Figures and Tables

**Table I.** Primer sequences of HPV-E7, RB, E2F1 and GAPDH.

Gene name		Sequence
HPV-E7	Forward	5′-ATGTCACGAGCAATTAAGC-3′
	Reverse	5′-TTCTGGCTTCACACTTACAACA-3′
RB	Forward	5′-CCTCCTTAATTTGGGAAGGTTTGTG-3′
	Reverse	5′-GCCTAACCCATAATGACCCTTGATT-3′
E2F1	Forward	5′-CAATCTGCACTTTGATTTGCTTCC-3′
	Reverse	5′-CCCGAAATGTTCCCAACAGA-3′
GAPDH	Forward	5′-ATGGGGAAGGTGAAGGTGG-3′
	Reverse	5′-GGGGTCATTGATGGCAACAATA-3′

HPV, human papilloma virus; RB, retinoblastoma.

## References

[1] Babjuk M (2010). New insights in intravesical treatment for intermediate- and high-risk non-muscle-invasive urothelial bladder carcinoma. Eur Urol.

[2] Shelley MD, Mason MD, Kynaston H (2010). Intravesical therapy for superficial bladder cancer: a systematic review of randomised trials and meta-analyses. Cancer Treat Rev.

[3] Brandau S, Riemensberger J, Jacobsen M (2001). NK cells are essential for effective BCG immunotherapy. Int J Cancer.

[4] Brandau S, Böhle A (2001). Activation of natural killer cells by Bacillus Calmette-Guérin. Eur Urol.

[5] Brandau S, Suttmann H, Riemensberger J (2000). Perforin-mediated lysis of tumor cells by *Mycobacterium bovis* Bacillus Calmette-Guérin-activated killer cells. Clin Cancer Res.

[6] Metawea B, El-Nashar AR, Kamel I, Kassem W, Shamloul R (2005). Application of viable bacille Calmette-Guérin topically as a potential therapeutic modality in condylomata acuminata: a placebo-controlled study. Urology.

[7] Böhle A, Büttner H, Jocham D (2001). Primary treatment of condylomata acuminata with viable bacillus Calmette-Guerin. J Urol.

[8] Fayed ST, Amer M, Ammar E, Salam MA (2009). Local BCG injection administered to patients with flat condyloma of the cervix. Int J Gynaecol Obstet.

[9] Singh S, Johnson J, Chellappan S (2010). Small molecule regulators of RB-E2F pathway as modulators of transcription. Biochim Biophys Acta.

[10] Cormio L, Tolve I, Annese P (2009). Altered p53 and pRB expression is predictive of response to BCG treatment in T1G3 bladder cancer. Anticancer Res.

[11] Esuvaranathan K, Chiong E, Thamboo TP (2007). Predictive value of p53 and pRB expression in superficial bladder cancer patients treated with BCG and interferon-alpha. Cancer.

[12] Thänhauser A, Böhle A, Flad HD, Ernst M, Mattern T, Ulmer AJ (1993). Induction of bacillus-Calmette-Guérin-activated killer cells from human peripheral blood mononuclear cells against human bladder carcinoma cell lines in vitro. Cancer Immunol Immunother.

[13] Suttmann H, Jacobsen M, Reiss K, Jocham D, Böhle A, Brandau S (2004). Mechanisms of bacillus Calmette-Guerin mediated natural killer cell activation. J Urol.

[14] Moretta A (1997). Molecular mechanisms in cell-mediated cytotoxicity. Cell.

[15] Münger K, Howley PM (2002). Human papillomavirus immortalization and transformation functions. Virus Res.

[16] Cobrinik D (2005). Pocket proteins and cell cycle control. Oncogene.

[17] Bremner R, Zacksenhaus E (2010). Cyclins, Cdks, E2f, Skp2, and more at the first international RB Tumor Suppressor Meeting. Cancer Res.

[18] Deshpande A, Sicinski P, Hinds PW (2005). Cyclins and cdks in development and cancer: a perspective. Oncogene.

[19] Chau BN, Wang JY (2003). Coordinated regulation of life and death by RB. Nat Rev Cancer.

[20] Ruiz S, Santos M, Segrelles C (2004). Unique and overlapping functions of pRB and p107 in the control of proliferation and differentiation in epidermis. Development.

[21] Wise-Draper TM, Wells SI (2008). Papillomavirus E6 and E7 proteins and their cellular targets. Front Biosci.

[22] Mitra AP, Birkhahn M, Cote RJ (2007). p53 and retinoblastoma pathways in bladder cancer. World J Urol.

